# Case Report: A case report and literature review of hemoglobin variation associated with neonatal cyanosis

**DOI:** 10.3389/fped.2024.1334757

**Published:** 2024-02-13

**Authors:** Yanru Chen, Jingwen Lv, Jihong Qian

**Affiliations:** Department of Neonatology, Xinhua Hospital Affiliated to Shanghai Jiaotong University School of Medicine, Shanghai, China

**Keywords:** neonatal cyanosis, anemia, neonatal outcome, hypoxia, whole-exome sequencing

## Abstract

We will discuss a recent case of unexplained neonatal cyanosis, evaluate its origin, clinical presentation, diagnosis, and treatment, and share with you some of our clinical insights. We report a transient cyanosis in a newborn due to a mutation in the globulin gene (HBG2), as well as diagnosis and treatment. Clinically, the infant was in good overall health, and despite low oxygen saturation, the arterial oxygen partial pressure was always normal. Early respiratory support includes mechanical ventilation, nasal tube oxygen, and eventually stopping oxygen therapy. With the above treatment measures, the blood oxygen saturation of the child always fluctuated at 85%, but the arterial blood oxygen partial pressure was up to 306 mmHg. Further improvement of laboratory tests revealed elevated methemoglobin levels, reticulocytosis, mild anemia, and basically normal on chest x-ray and echocardiography. To clarify the etiology, WES testing was performed. The results showed heterozygous variation in HBG2 gene (c.190C>T. p.H64Y). There is heterozygous variation at this site in the proband father, and no variation at this site in the proband mother. Given the age of the affected infants, we hypothesized that the mutation originated in the gamma peptide chain of the head protein. The baby was discharged from the hospital 10 days after birth, with blood oxygen saturation fluctuating around 90%. The cyanosis disappeared 2 months after discharge, and the blood oxygen saturation level returned to normal.

## Introduction

Neonatal cyanosis can be caused by mutations in the bead protein gene. In neonates with a mutation in the peptide gene, cyanosis is usually present in good general condition. The clinical features are consistently normal arterial partial pressure of oxygen despite hypoxemia and no combined pulmonary or cardiac disease. By summarizing the clinical features of transient neonatal cyanosis, we emphasize the role of genetic testing in the diagnosis and management of the disease, improve help in preventing misdiagnosis or missed diagnosis, and recommend genetic counseling for families with a relevant family history.

## Case report

She was a baby girl born by cesarean section at 37 5/7 weeks gestation. The APGAR score was 8 and 9 at 1 and 5 min, respectively. Birth weight was 3,160 g (27%) and head circumference was 33.5 cm (27%). Physical examination found that the baby's lips were blue and the whole body was cyanosis, but the muscle strength and tension of the limbs were normal and the primitive reflex was present.

**Figure 1:**
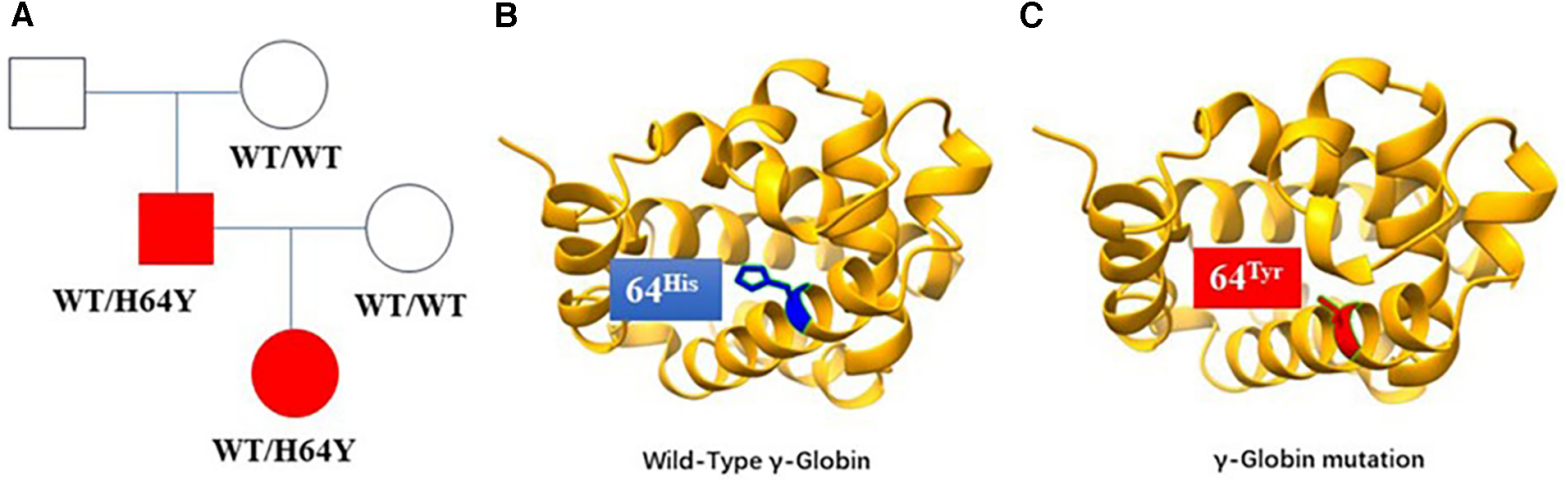
(**A**) Gamma-haemoglobin mutation lineages, with squares representing male members, circles representing female members, and shadows representing the case and her proband's father. (**B**) The schematic diagram of the wild-type structure of haemoglobin. (**C**) The predicted structure of the haemoglobin mutation in the patient. The heterozygous mutation of nucleotide 190 from cytosine C to thymine T (c.190C>T) resulted in the change of amino acid 64 from histidine to tyrosine.

Since the child's skin was bluish after birth and did not improve after oxygen and positive pressure ventilation, the initial percutaneous oxygen saturation measured in ambient air using a pulse oximeter was 78%–88%. After immediate mask oxygen administration, tracheal intubation and pure oxygen supply, the blood oxygen saturation of the child still fluctuated around 85%. The auxiliary examination showed no obvious abnormality in electrocardiogram, echocardiogram, and chest x-ray. Subsequently, the child was extubated and changed to nasal catheter oxygen inhalation until oxygen was stopped. The blood oxygen saturation fluctuated between 80% and 85%, and the arterial blood PaO2 reached a maximum of 306.7 mmHg. Laboratory tests showed a mild elevation of methemoglobin, mild anemia and reticulocytosis. We use vitamin C as a reductase enzyme to reduce the methemoglobin to hemoglobin and relieve cyanosis. We also infused red blood cell suspension to improve the anemia. The anemia was corrected and the methemoglobin level returned to normal, but the oxygen saturation still fluctuated around 90%. Clinical features are shown in Table 1. Whole-exome sequencing revealed a heterozygous mutation (c.190C>T p.H64Y) in the patient's HBG2 gene, which originated from the father but not the mother. (Laboratory: Beijing Maikino Medical Laboratory; Test: WES013: Trios). Figure 1 depicts a schematic illustration of a hemoglobin mutation. The case is discharged after 10 days and has a blood oxygen saturation of about 90%. The baby's cyanosis goes away in around two months, followed by physical health. The proband's father denied having experienced transient neonatal cyanosis.

**Table 1:** Clinical feature of the case.

Variable	Normal range	Postnatal days	
		PD 1	PD7
Oxygen saturation (%)	95–100	78↓	88↓
Hemoglobin (g/L)	145–220	134	145
Reticulocyte (%)	3–6	10.21↑	2.86
MCV (fl)	100–125	118.0	100.0
MCH (pg)	31–37	38.8	35.2
PaO2 (mmHg)	11–13	11.4	11.4
O2Hb (%)	92–98	87.4↓	92.8
Met Hb (%)	0–6	10.1↑	2.0
Lactate dehydrogenase (U/L)	0.7–2.1	3.8	2.9
Total bilirubin (μmol/L)	3–22	64.2↑	69.7↑
Chest x-ray	Normal	Normal	Normal
Echo-cardiogram	Normal	Normal	Normal

PD, denotes postnatal day.

## Discussion

Neonatal cyanosis can be caused by a variety of genetic and acquired causes, and mutations in the gamma-globin gene are an uncommon but important cause of neonatal cyanosis. It is clinically characterized by decreased hemoglobin oxygen saturation in infants without arterial hypoxemia and or a positive family history. We describe a case of cyanosis with neonatal onset, which developed immediately after birth and did not improve with oxygen therapy and intubation. However, blood gas results showed normal arterial partial pressure of oxygen, partial pressure of carbon dioxide and ph. His hemoglobin saturation fluctuated around 85%, and his arterial PaO2 reached 306.7 mmHg, and he was in good general condition. Laboratory tests showed mild anemia, mildly elevated methemoglobin, and elevated reticulocyte count; red blood cell transfusion was given to improve the anemia and vitamin C was given to lower the methemoglobin; the anemia was corrected and the methemoglobin returned to normal, but cyanosis did not improve, and the oxygen level of the child fluctuated around 85%. At the same time, auxiliary examinations showed that the echocardiogram, chest x-ray and electrocardiogram were normal.

To clarify the etiology of the disease, a WES test was performed; the results showed that the patient was heterozygous for the HBG2 gene (c.190C>T p.H64Y). This variant was initially recognized as pathogenic according to the American College of Medical Genetics and Genomics (ACMG) criteria, and the diagnosis was confirmed by the presence of a heterozygous variant in the father's locus and the absence of the variant in the mother's locus. The child was discharged from the hospital 10 days after birth with oxygen saturation fluctuating at 90%. The child was followed up until 2 months postnatally, when cyanosis disappeared and oxygen levels remained normal. WES is a relatively simple and rapid diagnostic protocol and is therefore used in clinical practice. Each hemoglobin in the human body is made up of four hemoglobin (also referred to as ferrous protoporphyrin) and a protein bead in the middle. Each hemoglobin is made up of four pyrrole subunits that form a ring with a ferrous ion in the center. Each of the four polypeptide chains in a bead protein, when connected to a hemoglobin, forms a monomer, or subunit, of hemoglobin (1). In an electrolyte solution resembling the setting in the human body, the four hemoglobin subunits can be automatically assembled into the α_2_β_2_ form. The four peptide chains that make up the bead proteins in adult and fetal hemoglobin are different; adults' hemoglobin can be consist of two α chains and two β chains, Hb A, whereas fetal hemoglobin may consist of two alpha and two gamma chains, Hb F. Fetal hemoglobin, which has a high oxygen-carrying capacity, is replaced shortly after birth by Hb A. The center of hemoglobin contains Fe^2+^, which can only be organized with oxygen when it binds to a porphyrin ligand; otherwise, it loses its ability to carry oxygen when it binds to cyanide or carbon monoxide. An intrinsic aspect of the protein structure has been an ancient relationship between the oxygen binding site and the multimerization interface, with surface modification dramatically lowering oxygen affinity and even conferring cooperativity. The interfaces between numerous amino acids that hold molecular complexes together typically involve sterically close, electrostatically complementary interactions (1). Here the two alpha chains combine with the gamma chain of hemoglobin (alpha-2/gamma-2), which is encoded by the HBG2 and HBG1 genes, to form fetal hemoglobin; both chains have an amino acid at codon 136, HBG1 has an alanine at codon 136, while HBG2 has a glycine. Considering the age of the sick baby, we can also surmise that the mutation comes from the γ peptide chain of the bead protein. No quantitative or functional studies of hemoglobin were performed to confirm the diagnosis of this child, and the lack of a comprehensive examination is regretted in this article. A limitation of our study is that, in this instance, we were unable to use high-performance liquid chromatography or protein electrophoresis to further test the child's hemoglobin; ordinarily, these procedures would be carried out prior to the genetic studies.

Five M-hemoglobin variants have been identified, two of which, Hb M Boston (Hisα58→Tyr) and Hb M Saskatoon (Hisα63→Tyr), have their distal histidine replaced by a tyrosine residue. In the neonatal period, hemoglobin is predominantly α- and γ-chained, whereas in the adult period it is predominantly α- and β-chained, and thus the hemoglobin chains affected by the same mutation may be different. Thus, the mutated gene in this case is consistent with Hb M Saskatoon, but the hemoglobin chain predominantly affected by the HBG mutation in the neonatal period is the γ chain. Additionally, a variety of variants mentioned in earlier literature are listed in Table 2 (2–9). The patient's hometown serves as the inspiration for the mutation name. Previous reports of hemoglobin variations causing transient newborn cyanosis include Hb F-M-Osaka [G 63(E7) His→Tyr], Hb F-M-Fort Ripley [G 92(F8) His→Tyr], Hb F Circleville [G 63(E7) His→Leu] and Hb F-M Toms River [G 67(E11) Val→Met] (2, 4, 7, 9, 10). A shift in the location between the subunits and a change in the shape of the tetramer are the outcomes of a mutation in the His of a hemoglobin's gamma subunit. Like in the instance, positions 63 and 92 of both the beta and gamma chains' amino acid residues are occupied, despite there being 39 differences between them. The imidazole group of the normal histidine residue does not form a bond with heme iron; however, the presence of the tyrosine residue results in the formation of a covalent linkage between its phenolic portion and heme iron, which stabilizes the heme iron in the Fe^3+^ form. In patients with the hemoglobin Toms River mutation, methionine replaces valine at E11, thereby preventing oxygen access to the iron atom in the center of the hemoglobin ring and destabilizing the hemoglobin tetramer, leading to hemolysis and anemia, and triggering cyanosis (2). When this occurs, O_2_ can no longer bind to Hb, and cyanosis occurs. The mutation that changed the 64th amino acid from histidine to tyrosine (p.H64Y) in the gamma-globin gene also contributed to our patient's altered hemoglobin. This mutation causes cyanosis by decreasing hemoglobin's affinity for oxygen.

**Table 2:** Known likely pathogenic or pathogenic variants of HBG2.

Variant ID(s)	Position(s)	Change	Clinical significance	PMID number
VAR_003166	93	H > Y	Pathogenic	2470017 (3)
VAR_003154	64	H > Y	Pathogenic	2483933 (4)
VAR_003146	42	F > S	Pathogenic	7741137 (6)
VAR_025336	64	H > L	Pathogenic	19065339 (9)
VAR_065950	68	V > M	Pathogenic	21561349 (2)
VAR_073159	106	L > H	Pathogenic	24502349 (8)

VAR, variant; PMID, pubmed unique identifier.

In conclusion, mutations in the gamma pearl protein gene are a rare but important cause of neonatal cyanosis. Clinical clues include reduced oxygen saturation in the absence of arterial hypoxemia, a positive family history, and an otherwise healthy infant with no evidence of cardiac or pulmonary disease. Definitive diagnosis by genetic testing can help provide accurate genetic and prognostic counseling and avoid missed diagnoses and misdiagnoses.

## Data Availability

The datasets presented in this article are not readily available because to preserve participant and patient anonymity. Requests to access the datasets should be directed to the corresponding author.
